# Multilocus haplotypes reveal variable levels of diversity and population structure of *Plasmodium falciparum *in Papua New Guinea, a region of intense perennial transmission

**DOI:** 10.1186/1475-2875-9-336

**Published:** 2010-11-23

**Authors:** Lee Schultz, Johanna Wapling, Ivo Mueller, Pilate O Ntsuke, Nicolas Senn, Joe Nale, Benson Kiniboro, Caroline O Buckee, Livingstone Tavul, Peter M Siba, John C Reeder, Alyssa E Barry

**Affiliations:** 1 Centre for Population Health, Burnet Institute, Melbourne, Australia; 2 Papua New Guinea Institute of Medical Research, Madang, Papua New Guinea; 3 Swiss Tropical and Public Health Institute, Basel, Switzerland; 4 Harvard School of Public Health, Boston, USA; 5 Monash University, Melbourne, Australia

## Abstract

**Background:**

The South West Pacific nation of Papua New Guinea has intense year round transmission of *Plasmodium falciparum *on the coast and in the low-lying inland areas. Local heterogeneity in the epidemiology of malaria suggests that parasites from multiple locations will need to be surveyed to define the population biology of *P. falciparum *in the region. This study describes the population genetics of *P. falciparum *in thirteen villages spread over four distinct catchment areas of Papua New Guinea.

**Methods:**

Ten microsatellite loci were genotyped in 318 *P. falciparum *isolates from the parasite populations of two inland catchment areas, namely Wosera (number of villages (n) = 7) and Utu (n = 1) and; and two coastal catchments, Malala (n = 3) and Mugil (n = 3). Analysis of the resultant multilocus haplotypes was done at different spatial scales (2-336 km) to define the genetic diversity (allelic richness and expected heterozygosity), linkage disequilibrium and population structure throughout the study area.

**Results:**

Although genetic diversity was high in all parasite populations, it was also variable with a lower allelic richness and expected heterozygosity for inland populations compared to those from the more accessible coast. This variability was not correlated with two proxy measures of transmission intensity, the infection prevalence and the proportion multiple infections. Random associations among the microsatellite loci were observed in all four catchments showing that a substantial degree of out-crossing occurs in the region. Moderate to very high levels of population structure were found but the amount of genetic differentiation (*F_ST_*) did not correlate with geographic distance suggesting that parasite populations are fragmented. Population structure was also identified between villages within the Malala area, with the haplotypes of one parasite population clustering with the neighbouring catchment of Mugil.

**Conclusion:**

The observed population genetics of *P. falciparum *in this region is likely to be a consequence of the high transmission intensity combined with the isolation of human and vector populations, especially those located inland and migration of parasites via human movement into coastal populations. The variable genetic diversity and population structure of *P. falciparum *has important implications for malaria control strategies and warrants further fine scale sampling throughout Papua New Guinea.

## Background

Malaria arising from infection with *Plasmodium falciparum *is a major cause of morbidity and mortality in tropical and sub-tropical regions of the world [1]. The difficulty in controlling this devastating disease has been due in part to high levels of genetic diversity of *P. falciparum*, allowing the rapid evolution and dissemination of advantageous traits such as drug resistance and antigenic variability. Malaria control would be more effective if the target parasite populations could be surveyed before an intervention to determine the extent of (i) genetic diversity, as a predictor of the populations' resilience to interventions; (ii) linkage disequilibrium, to understand the potential for multilocus haplotypes to spread through the region; and (iii) population structure, to map the distribution of diversity over geographic space and thus infer patterns of parasite migration. Population genetic surveys are therefore an essential preliminary step in designing the most appropriate and effective malaria control measures and as a baseline upon which to monitor their impact.

The worldwide population genetic structure of *P. falciparum*, as defined by multilocus genotyping, shows a general pattern of increasing genetic diversity, but decreasing linkage disequilibrium (LD) and population differentiation in association with the parasite transmission intensity (Americas < Asia Pacific <Africa) [2,3]. This original concept is being constantly updated, with new studies being used to describe the various patterns found locally within each continent. In the Americas, parasite populations continue to be characterized by low diversity, high levels of LD and strong population structure independent of geographic distance [4]. In Asia, more extensive studies have demonstrated higher levels of diversity than previously recognized and a lack of LD [5]. Moderate to high levels of population structure among countries of mainland Asia [6], and locations within Malaysia (Sumatra) and the Philippine islands [7,8] have been reported. In Africa, population structure, low levels of genetic diversity and significant LD have been described in the urban populations of Senegal, Niger and the Republic of Djibouti [9]. Significant LD has also been found in regions of high transmission and diversity in Senegal and the Republic of Congo [10,11]. The variable results observed are likely the result of inherent features to each geographic region such as the genetics and movement of human and anopheline hosts and biogeographical features that may interrupt gene flow, as well as the history of malaria transmission [2,12] and local malaria control efforts [7]. This emphasizes the importance of investigating the parasite population genetics within each region of interest, particularly now malaria elimination is back on the agenda in many countries.

The epidemiology of malaria in the South West Pacific nation of Papua New Guinea is highly variable. Malaria transmission is confined to the coastal and lowland zones where intense perennial transmission of the four major human malaria species (*P. falciparum, Plasmodium vivax, Plasmodium malariae *and *Plasmodium ovale*) occurs. Whereas, in the highlands, *Plasmodium *spp. infection is present but is mostly due to sporadic epidemic transmission [13]. In the endemic regions, the degree of *P. falciparum *transmission is high but variable among different regions, villages and even clusters of houses within villages [14], with entomological inoculation rates ranging from 0.15 - 1.44 infective bites/person/night [15-18]. Accordingly, the prevalence of multiple infections also varies greatly within this region, ranging from 26 to 50% [2,19-21], thus creating a range of opportunities for recombination between different genomes and the further generation of genetic diversity. This diverse micro-epidemiology has been attributed to differing patterns of host behaviour [22], nutrition [23-26], mosquito control [14,27], the wide range of vectors present [16,27,28] and more recently, bed net usage [20]. Variable patterns of parasite genetic diversity may result from, or underlie this mosaic pattern of malaria epidemiology. In addition, the biogeography of the country including mountains, thick forests and large rivers with limited transport have resulted in the isolation of human populations, as evidenced by the existence of more than 800 local languages [29] and different frequencies of human genetic polymorphisms that protect against malaria in different provinces [30]. The human diversity is matched by a highly diverse vector fauna with at least seven members of the *Anopheles punctulatus *complex and several minor species contributing to the transmission of malaria [13]. These vector species differ both in geographical distribution [27,31,32] and biting behaviour [33] and the major mosquito vector in the country, *Anopheles farauti s.l*. is known to fly only short distances (< 2 km). For these reasons, it is possible that gene flow among different parasite subpopulations of Papua New Guinea is limited. The presence of local parasite population structure has been suggested by differing seroprevalence to a *P. falciparum *antigen (S-antigen), even among closely spaced villages [34]. Consequently, it will be essential to sample more than one parasite population to explore the population structure of *P. falciparum *in Papua New Guinea. To date, there has only been one other study investigating the population biology of *P. falciparum *in Papua New Guinea by multilocus genotyping [2]. Using putatively neutral microsatellite markers, this study reported high levels of genetic diversity, a lack of LD and limited genetic differentiation between two neighbouring villages on the coast of Madang Province [2].

Given the locally variable epidemiology of malaria in Papua New Guinea [14], knowledge of the population genetics at different spatial scales is essential for decision-making for targeting *P. falciparum *populations for malaria control, managing the spread of drug resistance, developing approaches for vaccine design and eventually, elimination programs. The aim of this study was to describe the population genetics of *P. falciparum *sampled from thirteen villages spread over four distant catchments of East Sepik and Madang Provinces. Multilocus haplotypes were defined by genotyping a validated panel of microsatellite markers [35] and the extent and distribution of genetic diversity within and among parasite populations was measured. The results have important implications for programs targeted at controlling and eliminating this major human pathogen.

## Methods

### Study sites and *P. falciparum *isolates

**Figure 1:**
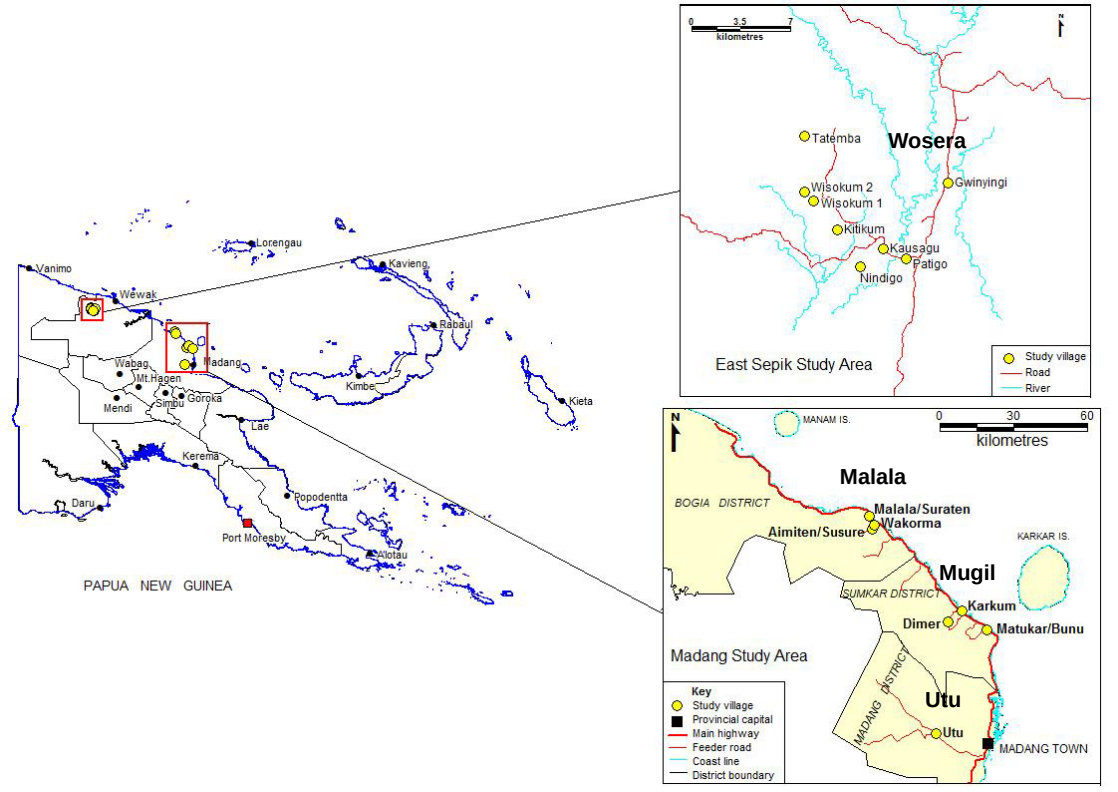
**Map of the study sites**.

East Sepik and Madang Provinces have long been the focus of malaria research and ongoing control efforts in Papua New Guinea. To represent a broad cross-section of the targeted parasite populations and to limit bias in the dataset, venous blood samples were collected from asymptomatic human volunteers of all ages in cross-sectional malaria surveys. In East Sepik Province, the survey took part in August and September of 2005 with 872 samples collected from individuals residing in one catchment area including seven villages spaced between 2-10 km apart in the Wosera (Gwinyingi, Patigo, Nindigo, Kitikum, Wisokum (1 and 2) and Tatemba) (Figure 1). In Madang Province, the survey took place in March 2006 with 1,275 samples collected from individuals residing in three distinct catchment areas including ten villages within 5-20 km of Mugil (Dimer, Karkum and Matukar/Bunu), Malala (Amiten/Susure, Malala/Suraten and Wakorma) and Utu (Utu) health centres (Figure 1). As samples from three pairs of villages in Mugil and Malala were combined, and the Utu villages were collected during single surveys of three nearby villages, the samples represent the parasite populations of a total of thirteen villages or neighbouring village-pairs. For simplicity "village" is used throughout the manuscript. Ethical approval to conduct the study was granted by the PNG Institute of Medical Research Institutional Review Board, the Papua New Guinea Medical Research Advisory Committee and the Alfred Hospital Research and Ethics Unit.

Genomic DNA was extracted from whole blood samples using the 96 well QiaQuick DNA extraction kit (Qiagen). To identify *P. falciparum *positive samples and the number of infecting clones concurrently, the highly polymorphic antigen gene *msp2 *was genotyped as previously described [36]. This approach utilizes a nested multiplex PCR to simultaneously amplify *msp2 *from genomic DNA with different fluorescent primers specific for 3D7 and FC27 allele families. Agarose gel electrophoresis identified samples with a positive PCR result and thus *P. falciparum *infection. Fluorescently labelled PCR products were then analysed with an ABI capillary electrophoresis platform with the internal size standard GS LIZ500 (Applied Biosystems). Resultant chromatograms were analysed with Peak Scanner V1.0 software (Applied Biosystems) to count the number of 3D7- and FC27-specific peaks (alleles) and thus estimate the total number of *P. falciparum *clones. From this we calculated two molecular epidemiological correlates of *P. falciparum *transmission intensity for each village: (i) the infection prevalence, calculated as the proportion (%) of samples with a positive PCR result and (ii) the proportion of infections with multiple clones, defined by the presence of more than one peak (allele) on the chromatogram. Only the *P. falciparum *isolates shown to contain single *msp2 *alleles were used for microsatellite genotyping.

### Microsatellite genotyping

Due to the low parasitaemia and limited quantity of the field samples, whole genome amplification of the selected *P. falciparum *isolates was performed using the Illustra Genomiphi V2 DNA amplification kit (GE Healthcare) according the manufacturer's instructions. Each of these isolates were then genotyped using ten putatively neutral microsatellite markers developed by Anderson and colleagues ([35] TA1, TA60, Polyα, ARA2, Pfg377, TAA87, TAA42, PfPK2, TAA81 and 2490) with a reduced primer concentration of 0.08 mM. Fluorescently labelled PCR products were visualized with an ABI capillary electrophoresis platform and resultant chromatograms analysed using Peak Scanner V1.0 software (Applied Biosystems) to define alleles. Several isolates showed multiple peaks (alleles) with secondary peaks having a height greater than 30% that of the predominant peak, indicating the presence of multiple clones [35]. This was expected because some clones will share *msp2 *alleles and thus can only be distinguished by genotyping at additional loci. True "single infections" were defined as those containing only one allele for all microsatellite loci and those with two alleles at only one locus. The latter was a precaution against genotyping artefacts or within-clone variation that may result in two or more visible peaks on the chromatogram. "Multiple infections" were, therefore, defined as those in which at least two loci contained multiple alleles. Following the methodology of Anderson *et al *[35], multiple infections with only two alleles were included in the dataset by reconstructing haplotypes from the predominant peaks for each locus. All isolates with more than two alleles at any locus were excluded. At least 75% of the isolates were genotyped successfully for each of the ten loci.

### Population genetic analysis

Allele frequencies for the 13 villages and overall were determined using CONVERT version 1.31 software. This software was then used to generate input files for the various population genetic software used [37]. Genetic diversity was assessed using ARLEQUIN version 3.11 software [38] by determining the number of haplotypes (*h*), the number of alleles per locus (*A*) and the expected heterozygosity, calculated as $H_{e}=\frac{n}{n-1}(1-\sum_{i=1}^{n}p_{i}^{2})$, where *p *is the frequency of the *i*^th ^allele and *n *is the number of alleles in the sample. Because *A *is strongly influenced by sample size it is only reliable for large sample sizes (e.g. catchments) therefore we also calculated the allelic richness (*R_s_*) which is normalized on the basis of the smallest sample size and based on the rarefaction method developed by Hurlbert [39] and implemented in FSTAT version 2.9.3 software [40]. Associations between the latter two diversity indices and correlates of transmission intensity were measured by Spearmans rank correlation test using SPSS version 17. To measure multilocus LD (non-random associations among loci), the standardized index of association (*I^S^_A_*) was calculated using the program LIAN version 3.5 [41] for the whole dataset and a curtailed dataset with haplotypes only from confirmed single infections, as a precaution against the bias that may result from presence of any false dominant haplotypes [2]. As only complete haplotypes could be analysed by LIAN version 3.5, to maximize sample size, this analysis included only eight loci (TA1 and TAA42 were excluded). Due to the small size of the dataset within some villages, LD was calculated only on the scale of each catchment. Population differentiation was estimated by using two pairwise distance measurements: *F_ST _*(*θ*, which estimates the weighted average F statistics over all loci based on the number of different alleles between haplotypes [42]; and *R*_ST _which calculates F statistics from the sum of the squared size difference (i.e. number of repeat units) between haplotypes [43] using only the seven microsatellite loci that follow the simple step-wise mutation model (TA87, ARAII, Pfg377, 2490, TA81, PfPK2 and TA60; [44]).

Significance for both *F_ST _*and *R_ST _*was tested by comparison with 95% confidence intervals from 1023 permutations. As *R_ST _*considers the distances between alleles it is the more sensitive of the two statistics. Correlations between genetic differentiation and geographic distance (the shortest distance in km, as defined by the exact distance between geographic co-ordinates) were measured using the Mantel test [45] in FSTAT version 2.9.3 [40]. As small sample size may result in a biased estimate of genetic differentiation the Mantel tests included only villages with n ≥ 22. To confirm the population structure identified by F statistics, *Structure *v. 2.3 software [46] was also used to test whether each haplotype clustered according to geographic origin. *Structure *assigns individual multilocus haplotypes probabilistically to one of a number of clusters (*K*) or jointly to multiple clusters (admixture) based on the allele frequencies at each locus [46,47]. The analysis was run 20 times for *K *= 1-20 for 10,000 Monte Carlo Markov Chain (MCMC) iterations after a burn-in period of 10,000 using the admixture model and correlated allele frequencies for the analysis. The most likely *K *was defined by calculating the rate of change of *K*, ΔK, according to the method of Evanno *et al *[48] and geographic population structure determined by assessing whether the ancestry coefficients were asymmetric among sampling locations [47]. To further visualize the complex relationships among haplotypes that might result from recombination a weighted network approach that connects haplotypes if they shared at least three alleles was utilized. Network analysis was done using the free software Cytoscape [49]. Each node within the network represents an individual haplotype, and edges between nodes represent shared alleles between haplotypes. For visual clarity, a threshold was set such that nodes were only joined by edges if they shared more than three loci. Modifications of this threshold value did not qualitatively change the structure of the network. Above this threshold, the edges in the network were weighted according to the number of shared alleles. Missing data points were assumed to be different between loci. An edge-weighted spring-embedded algorithm was used to construct the network. Based on Kamada and Kawai's notion of "force-directed" networks [50], the algorithm treats nodes as objects that repel each other dependent on a spring force between them, which is modified by the weight of the edge.

## Results

From a total of 2147 samples, *msp2 *genotyping identified 765 *P. falciparum *isolates. Throughout the study area there was considerable variation in the infection prevalence (village: 12-47%; catchment: 28-44%) and the proportion of isolates that contained multiple clones (village: 0-65%; catchment: 39-45%) (Additional file 1) indicating a broad range of transmission intensities throughout the study area. Of the *P. falciparum *isolates, 431 (Wosera = 142, Utu = 87, Malala = 82, Mugil = 120) contained single *msp2 *alleles and were thus selected for genotyping at ten microsatellite loci. Microsatellite genotyping confirmed 213 single clone infections (Wosera = 87, Utu = 45, Malala = 42, Mugil = 38) while revealing a further 219 multiple clone infections including 106 with only two clones (Wosera = 25, Utu = 21, Malala = 26, Mugil = 34). Haplotypes were reconstructed from the single clone infections (single haplotypes) and two clone infections (dominant haplotypes) as described in detail in the Materials and Methods. The remaining 113 samples were found to contain more than two clones and were excluded from the dataset. There was no significant genetic differentiation between single and dominant haplotypes within each catchment (Wosera: *F*_ST _= 0.015, *P *= 0.34, Utu: *F*_ST _= 0.009, *P *= 0.81; Malala: *F*_ST _= 0.036, *P *= 0.02; Mugil: *F*_ST _= -0.019, *P *= 0.99;) so the two datasets were combined, giving a total of 318 multilocus haplotypes for population genetic analysis (Table 1). Large sample sizes were available for analysis by catchment (n = 66-112) and for the majority of villages (n = 22- 66) with only small sample sizes (n ≤ 14) available for Wisokum, Patigo, Kitikum, Tatemba Malala/Suraten and Dimer (Table 1).

**Table 1:** Genetic diversity in Plasmodium falciparum populations of Papua New Guinea

Population	*n^a^*	*h^b^*	*A^c ^± SE*	*R_s_^d ^± SE*	*H_e_^e ^± SE*
					
***Wosera***	***112***	***112***	***11.18 ± 0.28***	***9.65 ± 0.78***	***0.73 ± 0.01***
Gwinyingi	23	23	5.82 ± 0.38	4.60 ± 0.42	0.68 ± 0.04
Patigo	13	13	4.55 ± 0.38	4.21 ± 0.38	0.67 ± 0.06
Nindigo	38	38	6.64 ± 0.27	4.42 ± 0.34	0.69 ± 0.02
Kitikum	13	13	5.00 ± 0.37	4.60 ± 0.33	0.76 ± 0.03
Wisokum	14	14	4.73 ± 0.45	4.08 ± 0.42	0.65 ± 0.05
Tatemba	11	11	5.27 ± 0.41	4.70 ± 0.35	0.75 ± 0.04
					
***Utu***	***66***	***66***	***7.27 ± 0.29***	***7.30 ± 0.69***	***0.64 ± 0.03***
Utu	66	66	7.27 ± 0.29	4.29 ± 0.41	***0.64 ± 0.03***
					
***Malala***	***68***	***68***	***9.54 ± 0.30***	***8.57 ± 0.75***	***0.77 ± 0.08***
Amiten/Susure	22	22	6.82 ± 0.40	5.07 ± 0.32	0.76 ± 0.02
Malala/Suraten	12	12	4.64 ± 0.43	4.27 ± 0.42	0.73 ± 0.04
Wakorma	34	34	7.54 ± 0.36	5.13 ± 0.34	0.77 ± 0.01
					
***Mugil***	***72***	***72***	***9.36 ± 0.23***	***9.21 ± 0.63***	***0.76 ± 0.01***
Dimer	16	16	5.36 ± 0.38	4.58 ± 0.3	0.75 ± 0.02
Karkum	28	28	6.64 ± 0.31	4.84 ± 0.38	0.73 ± 0.03
Matukar/Bunu	28	28	6.73 ± 0.28	4.95 ± 0.28	0.76 ± 0.02
					
**TOTAL**	**318**	**318**	**14.00 ± 0.21**	**10.58 ± 0.76^f^**	**0.79 ± 0.01**

^a ^number of isolates genotyped; ^b ^number of haplotypes; ^c ^mean number of alleles per locus; ^d ^allelic richness; ^e ^expected heterozygosity; ^f ^calculated based on minimum catchment size

### Genetic diversity

There were as many haplotypes (*h*) as isolates successfully genotyped (*n*) in the dataset showing that all haplotypes were unique (Table 1). The inland catchment of Utu had the lowest mean number of alleles (A), allelic richness (*R_s_*) and expected heterozygosity (*H_e_*). Because it had a larger sample size, Wosera had the highest *A *but the normalized statistic *R_s, _*was similar to that of Mugil and Malala and it had the second lowest *H_e _*after Utu. For the villages, Utu and several of the Wosera villages had the lowest values for all diversity parameters compared to the majority of coastal villages (Table 1). It should be noted that the different *R_s _*values observed for Utu considered as either a catchment or village were the result of recalculation on the basis of the smallest sample size [39]. In contrast to the inland parasite populations, the coastal catchments of Mugil and Malala and the villages within them showed some of the highest values for all diversity parameters. In addition to the variable levels of diversity observed among catchments, *R_s _*and *H_e _*were highly variable within the catchments of Malala and the Wosera (Table 1). There was no significant correlation between genetic diversity (*R_s _*and *H_e_*) and the correlates of transmission intensity (Additional file 2). Less diversity was observed within villages compared to the catchments and also within catchments compared to the total (note that it was only possible to compare *H_e _*between the two scales). In addition, allele frequencies varied among sites (Additional file 3). Higher levels of diversity among compared to within populations and differing allele frequencies between populations indicate the presence of population structure within the study area.

### Multilocus linkage disequilibrium

Non-random associations among loci (multilocus LD) were measured for all complete haplotypes (n = 159) and also those from single infections (n = 111) by calculating the Index of Association (*I_A_^S^*). The latter analysis was used to confirm LD in the absence of haplotypes predicted from multiple infections, which can result in higher estimates of recombination and thus bias against the detection of LD. To check whether associations may have arisen from clonal propagation, LD can be measured among unique haplotypes [51], but this was not necessary because all haplotypes in the dataset were unique (Table 1). Consistent with the high proportion of multiple infections in all populations (Additional file 1), no significant LD was identified within any of the catchments for both the full dataset and for the single infections, but LD was significant when all catchments were combined (total; Table 2).

**Table 2:** Multilocus linkage disequilibrium in Plasmodium falciparum populations of Papua New Guinea

*Population*	All Infections		Single Clones	
	n^a^	*I^S^_A _*(p-value)	n^a^	*I^S^_A _*(p-value)
				
***Wosera***	38	-0.0049 (0.672)	32	-0.0076 (0.718)
***Utu***	52	0.0013 (0.422)	36	0.0033 (0.352)
***Malala***	34	0.0015 (0.392)	22	0.0074 (0.708)
***Mugil***	35	0.0013 (0.426)	21	0.0044 (0.377)
				
**TOTAL**	**159**	**0.0088 (0.010)**	**111**	**0.0046 (0.147)**

^a^Number of isolates

### Population structure

The calculation of population pairwise differentiation using both *F*_ST _and *R*_ST _showed significant differentiation among the catchment areas (Table 3). The strongest differentiation was observed between the inland and coastal catchments, and the weakest between the coastal catchments and between Malala and Wosera. Pairwise analysis of the differentiation between villages provided further insight into the structure among catchments and identified significant pairwise values between villages of Malala, Mugil and Wosera (Additional file 4). Mantel tests showed no significant correlation between either of the two measures of genetic differentiation and geographic distance (Additional file 5).

**Table 3:** Genetic differentiation between Plasmodium falciparum populations of Papua New Guinea

	WOSERA	UTU	MALALA	MUGIL
**WOSERA**		0.08**	0.05**	0.08**
**UTU**	0.14**		0.16**	0.09**
**MALALA**	0.05**	0.11**		0.06**
**MUGIL**	0.09**	0.12**	0.06**	

Matrix of pairwise *F*_ST _(lower diagonal) and *R*_ST _values (upper diagonal) between catchments

**Figure 2:**
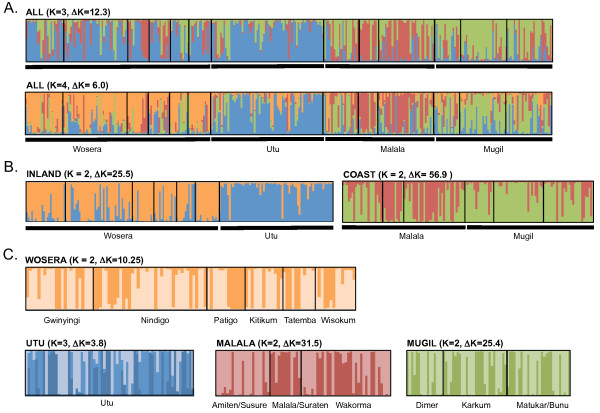
***Structure *analysis of 318 *Plasmodium falciparum *microsatellite haplotypes from Papua New Guinea**. Individual ancestry coefficients for A) all haplotypes for *K *= 3 and K = 4, B) inland and coastal datasets and C) catchments. Each bar represents the proportion of each haplotype with ancestry in the defined clusters, each cluster being indicated by a different colour. Black borders around groups of haplotypes represent the different villages. The number of clusters (*K*) associated with each plot is indicated along with the rate of change of *K *(ΔK).

The cluster analysis for the full dataset initially defined three clusters (i.e. the highest value of ΔK occurred at K = 3; Additional file 6). For this distribution, Utu and Wosera haplotypes were predominantly assigned to the same cluster (Figure 2A) possibly because of the weaker population structure between the two inland populations than that between inland and coastal populations (Table 3). Confirming this, separate analyses for the inland and coast haplotypes clearly defined two distinct populations for each dataset (ΔK peaked at K = 2; Additional file 6) with the majority of haplotypes from different catchments assigned to different clusters (Figure 2B). Therefore, the distribution of all haplotypes among four clusters more appropriately summarizes the geographic population structure in Papua New Guinea (K = 4, Figure 2A). To further investigate the possibility of weak local population structure within catchments, we also reran the analysis separately for each catchment. This revealed two clusters for all catchments except Utu, which had three clusters (ΔK peaked at K = 2 for Wosera, Mugil and Malala but at K = 3 for Utu; Additional file 6). However, the clustering patterns were relatively symmetric among villages, except for the Malala catchment in which Amiten/Susure haplotypes were assigned predominantly to only one cluster (Figure 2C). For the larger datasets Amiten/Susure haplotypes were predominantly assigned to the same cluster as those from Mugil (Figures 2A and 2B). Therefore, the cluster analysis confirmed geographic population structure among catchments and within the Malala catchment. The population structure detected within other catchments by this analysis was not a consequence of the spatial separation of parasite populations.

**Figure 3:**
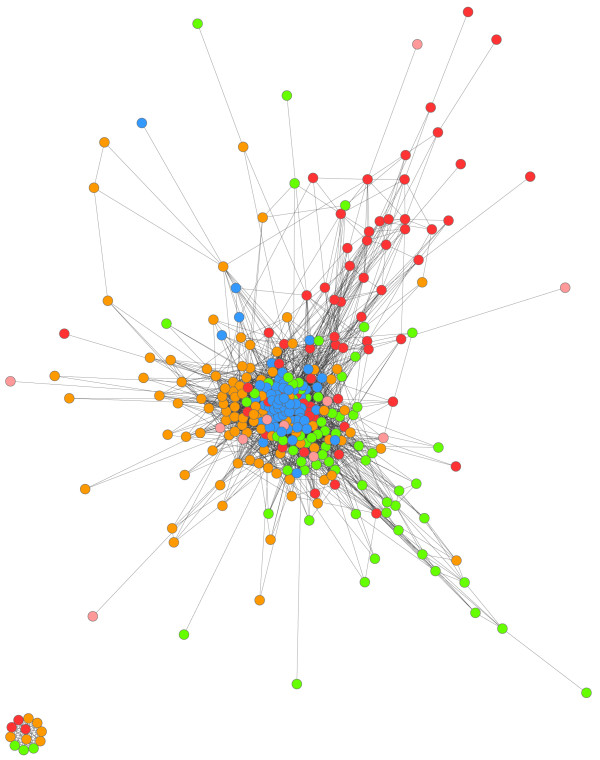
**Network analysis of 318 *Plasmodium falciparum *microsatellite haplotypes from Papua New Guinea**. Weighted network of haplotypes showing relationships among individuals of each catchment. Each coloured circle represents a haplotype (node), and black lines indicate shared alleles among individual haplotypes. Orange = Wosera, Blue = Utu, Red = Malala, Green = Mugil. The threshold for a connection was set at three matching loci between two haplotypes.

Confirming the above analyses of population structure between catchments, the network shows that the majority of connections were between haplotypes from the same catchment (Figure 3). The Utu haplotypes formed a densely connected central cluster with few weakly linked nodes consistent with the lower diversity in the catchment, whereas the more diverse Wosera, Malala and Mugil haplotypes were more loosely connected to each other but formed separate lobes of the network radiating from the centre (Figure 3). The tightly connected Utu haplotypes can be explained by the presence of high frequency alleles for three loci (TAA109, TAA42, 2490; Additional file 3). The smaller peripheral network containing haplotypes from Wosera, Mugil and Malala indicates a panel of related haplotypes that shared fewer than three alleles with any of the haplotypes in the main network (Figure 3). Individual networks for each catchment consisted of a single lobe, with connections both within and among villages arguing against the presence of population structure (Additional file 7). For the Malala catchment though, the majority of Amiten/Susure haplotypes were more closely connected at the top of the network consistent with the *F_ST _*and cluster analyses.

## Discussion

This is the most extensive study to date investigating the genetic structure of *P. falciparum *populations of Papua New Guinea. Included in the study were the parasite populations of thirteen villages (or village-pairs) distributed over two inland and two coastal catchment areas in the north of the country where malaria research and control efforts are focused. A previous analysis of the same set of microsatellite loci in two coastal villages (Buksak and Mebat) approximately 80 km apart in nearby areas of Madang Province reported a high degree of genetic diversity (*H*_e _= 0.62 - 0.65), a lack of significant LD (*I_S_^A ^*= 0.0055- 0.0073; *P *> 0.05) and minimal differentiation between the two populations (*F*_ST _= 0.015) [2]. We have similarly identified high levels of diversity and a lack of LD, however by surveying many more villages over a larger area, we have discovered a wider range of diversity than previously shown (*H*_e _= 0.64-0.77) and that parasite populations are heterogeneous with moderate to very high population structure detected throughout the study area (*F*_ST _= 0.05 - 0.33; *P *< 0.01). Differences between the study of Anderson *et al *[2] and the current findings are consistent with a variety of *P. falciparum *population structures throughout Papua New Guinea.

Levels of diversity are an indication of the fitness of the parasite population and thus how difficult it may be to target with drugs or vaccines. The diversity among catchments was high but also variable, with the inland populations of Wosera and Utu having lower levels of allelic richness and heterozygosity than the coastal populations of Malala and Mugil. The lack of association between the molecular epidemiological correlates of transmission and diversity in Papua New Guinea suggest that a number of factors influence the population genetics of *P. falciparum *in the region, these are discussed below. Given the potential difficulty in controlling diverse parasites, such knowledge has important practical implications for malaria control across the country.

As Papua New Guinean parasite populations had high levels of genetic diversity it was not surprising to find a lack of significant LD in all four catchments studied, while the LD found for the total dataset can be explained by the Wahlund effect due to the observed population structure [52]. Within each parasite population a large proportion of multiple infections was found and therefore cross-fertilisation and recombination between distinct parasite genomes would be expected to maintain random associations among loci. LD has important implications for the spread of multilocus drug resistance haplotypes, with high levels of inbreeding increasing their dispersal. In Papua New Guinea, the lack of LD combined with the geographic population structure would be unlikely to facilitate such events.

The population structure between the inland (Wosera and Utu) and coastal populations (Malala and Mugil) indicated the existence of barriers to gene flow and thus parasite migration, and other possible influences on population structure such as natural selection and genetic drift within each catchment. Although only ~50 km from the provincial capital of Madang town, it takes several hours to travel to the remote Utu village with only one road entering and leaving and there is no direct route of travel (road or air) between Madang and the East Sepik Provinces. Whereas, the lower extent of population structure between Malala and Mugil, and small amount of mixing between these two populations indicated in the cluster and network analyses probably reflects their direct connection via the Pacific Highway. Despite this direct route of possible gene flow between Malala and Mugil, the population structure was significant. Migration of diverse parasites into these locations via human movement may partially explain this observation. For Malala, there is a boarding school with students attending from across Madang and East Sepik Provinces. Lower levels of differentiation among catchments occurred between Wosera and Malala so it is plausible that some gene flow occurs between these locations via movement of students and their guardians. In Mugil, there is constant movement of people ferrying to and from the well-populated Karkar Island (17 km of the coast), however the lack of samples from this location makes this speculation difficult to confirm. The genetic differentiation and cluster analyses also indicated that population structure occurs on a local scale (< 20 km). In particular, in the Malala area the village of Amiten/Susure which is located ~6 km inland was found to be genetically distinct from Malala/Suraten and Wakorma which flank the school. In fact, these analyses suggested that the Amiten/Susure population was more similar to parasites from the Mugil villages, suggesting that they may represent the "true Madang coast" population. In Mugil and Wosera, some villages were also differentiated. Here, the low but significant *F_ST _*values can be explained by small sample sizes for all except for that between Matukar/Bunu and Karkum (Mugil), and Nindigo and Gwinyingi (Wosera). However, the cluster and network analyses did not suggest any geographic population structure within catchments other than Malala. An isolation-by-distance model did not explain the observed geographic population structure, suggesting that there is a non-continuous distribution of diversity in Papua New Guinea. This fragmented population structure may be explained by movement of the human host with a lack of transport between catchments combined with higher rates of migration into coastal catchments as described above.

There are other possible explanations for the patterns of population structure within Papua New Guinea. People from different catchments belong to different language groups [29], indicating historical separation of human populations and presumably the parasites infecting them. A different prevalence of genetic polymorphisms that protect against malaria between Madang and the East Sepik [30] might have also provided unique selective pressures for the respective parasite populations. In addition, a possible role for the anopheline vector in shaping the observed population genetic structure of *P. falciparum *in Papua New Guinea cannot be ignored. At least six distinct anophelene species transmit malaria in the region. In Madang Province, *Anopheles farauti *4 is predominant in the inland villages such as Utu and Amiten/Susure whereas *A. faurauti *1 is more common in villages that are proximal to the coast [31,32]. In the Wosera, *Anopheles koliensis *and *Anopheles punctulatis *are the predominant vectors [27]. The population structure observed was consistent with these vector distributions. In Africa, investigators have found no evidence of *P. falciparum *population structure between two co-existing vectors, *Anopheles gambiae *and *Anopheles funestus *[53] suggesting that transmission by these vector species is not a strong barrier to gene flow. In Papua New Guinea though, the vector species distribution is highly heterogeneous with a limited overlap [27,31,32] so the ability of the different species to transmit allopatric parasites would have to be tested. Other factors that influence transmission intensity such as the use of bed nets [20] may also impact on the overall population structure. Whatever the explanation, the population structure of *P. falciparum *in Papua New Guinea is likely the result of a combination of factors, including the limited movement of both human and mosquito hosts, in addition to the greater accessibility of the coast in comparison to the inland populations. The public health implication for these findings is that parasite populations that might be assumed to be similar for development of malaria control strategies, such as vaccines, in fact are genetically distinct and thus may respond differently to such interventions. However, some populations may be easier to control if they are isolated from external sources of parasites. Utu, having the least diverse and most genetically differentiated parasite population appears to be the most isolated catchment, and thus a location where malaria control strategies may be the most efficient.

## Conclusions

A detailed understanding of the population genetics of *P. falciparum *can help guide malaria control efforts. Such knowledge is becoming paramount as the Papua New Guinean government prepares to intensify malaria control, not only for guiding these control efforts but also monitoring whether they are having an impact on parasite populations. This broad spatial survey of the population genetics of *P. falciparum *in Papua New Guinea has identified high but variable levels of genetic diversity, random associations among loci and population structure found at different spatial scales. The results have significant implications for malaria control in the Pacific region and show that countrywide population surveillance is needed throughout Papua New Guinea.

## Competing interests

The authors declare that they have no competing interests.

## Authors' contributions

L.S. performed experiments, data analysis and helped write the paper. J.W. and P.O.N. performed experiments and data analysis. I.M. co-ordinated the field studies, provided *P. falciparum *samples and helped interpret the results. N.S., J.N., B.K. and L.T. co-ordinated the field studies and collected samples. C.O.B. performed the network analysis. P.M.S. provided logistical support. J.C.R. helped co-ordinate the study and write the paper. A.E.B. conceived the study design, co-ordinated the study, performed experiments and data analysis and wrote the paper. All authors read and approved the final manuscript.

## Supplementary Material

Additional file 1 **Intensity of *P. falciparum *transmission in Papua New Guinea**. Two correlates of parasite transmission, the infection prevalence and the proportion of infected people carrying multiple *P. falciparum *clones, were estimated by *msp2 *genotyping. Click here for file

Additional file 2 **Associations between transmission intensity and genetic diversity in Papua New Guinea**. Matrix of Spearmans rank correlation coefficients (ρ) and in brackets, associated p-values. Click here for file

Additional file 3 **Microsatellite allele frequencies for *Plasmodium falciparum *populations from Papua New Guinea**. n/a. Click here for file

Additional file 4 **Genetic differentiation between *Plasmodium falciparum *populations of Papua New Guinea**. Matrix of pairwise *F*_ST _(lower diagonal) and *R*_ST _values (upper diagonal) between villages. Click here for file

Additional file 5 **Associations between geographic distance (km) and pairwise genetic differentiation in Papua New Guinea**. Matrix of Mantel correlation results and in brackets, p-values. Click here for file

Additional file 6 **Definition of the most probable number of clusters for 318 *Plasmodium falciparum *microsatellite haplotypes from Papua New Guinea**. *Structure *analysis (ΔK plots) for A) all haplotypes (B) inland and coastal datasets and (C) and each of the catchments. Click here for file

Additional file 7 **Network analysis of 318 *Plasmodium falciparum *haplotypes within four catchment areas of Papua New Guinea**. Weighted network of haplotypes showing relationships among individuals for each catchment (A) Wosera (B) Utu (C) Malala and (D) Mugil. Each coloured circle represents a haplotype (node), and black lines indicate shared alleles among individual haplotypes. The threshold for a connection was set at three matching loci between two haplotypes. Click here for file

## References

[B1] Snow RW Guerra CA Noor AM Myint HY Hay SI The global distribution of clinical episodes of *Plasmodium falciparum *malaria Nature 2005 434 214 217 10.1038/nature03342 15759000 PMC3128492

[B2] Anderson TJ Haubold B Williams JT Estrada-Franco JG Richardson L Mollinedo R Bockarie M Mokili J Mharakurwa S French N Whitworth J Velez ID Brockman AH Nosten F Ferreira MU Day KP Microsatellite markers reveal a spectrum of population structures in the malaria parasite *Plasmodium falciparum* Mol Biol Evol 2000 17 1467 1482 11018154 10.1093/oxfordjournals.molbev.a026247

[B3] Mu J Awadalla P Duan J McGee KM Joy DA McVean GA Su XZ Recombination hotspots and population structure in *Plasmodium falciparum* PLoS Biol 2005 3 e335 10.1371/journal.pbio.0030335 16144426 PMC1201364

[B4] Machado RL Povoa MM Calvosa VS Ferreira MU Rossit AR dos Santos EJ Conway DJ Genetic structure of *Plasmodium falciparum *populations in the Brazilian Amazon region J Infect Dis 2004 190 1547 1555 10.1086/424601 15478058

[B5] Pumpaibool T Arnathau C Durand P Kanchanakhan N Siripoon N Suegorn A Sitthi-Amorn C Renaud F Harnyuttanakorn P Genetic diversity and population structure of *Plasmodium falciparum *in Thailand, a low transmission country Malar J 2009 8 155 10.1186/1475-2875-8-155 19602241 PMC2722663

[B6] Mu J Myers RA Jiang H Liu S Ricklefs S Waisberg M Chotivanich K Wilairatana P Krudsood S White NJ Udomsangpetch R Cui L Ho M Ou F Li H Song J Li G Wang X Seila S Sokunthea S Socheat D Sturdevant DE Porcella SF Fairhurst RM Wellems TE Awadalla P Su XZ *Plasmodium falciparum *genome-wide scans for positive selection, recombination hot spots and resistance to antimalarial drugs Nat Genet 2010 42 268 271 10.1038/ng.528 20101240 PMC2828519

[B7] Anthony TG Conway DJ Cox-Singh J Matusop A Ratnam S Shamsul S Singh B Fragmented population structure of *Plasmodium falciparum *in a region of declining endemicity J Infect Dis 2005 191 1558 1564 10.1086/429338 15809916

[B8] Iwagami M Rivera PT Villacorte EA Escueta AD Hatabu T Kawazu S Hayakawa T Tanabe K Kano S Genetic diversity and population structure of Plasmodium falciparum in the Philippines Malar J 2009 8 96 10.1186/1475-2875-8-96 19422722 PMC2685811

[B9] Bogreau H Renaud F Bouchiba H Durand P Assi SB Henry MC Garnotel E Pradines B Fusai T Wade B Adehossi E Parola P Kamil MA Puijalon O Rogier C Genetic diversity and structure of African *Plasmodium falciparum *populations in urban and rural areas Am J Trop Med Hyg 2006 74 953 959 16760503

[B10] Durand P Michalakis Y Cestier S Oury B Leclerc MC Tibayrenc M Renaud F Significant linkage disequilibrium and high genetic diversity in a population of *Plasmodium falciparum *from an area (Republic of the Congo) highly endemic for malaria Am J Trop Med Hyg 2003 68 345 349 12685643

[B11] Leclerc MC Durand P de Meeus T Robert V Renaud F Genetic diversity and population structure of Plasmodium falciparum isolates from Dakar, Senegal, investigated from microsatellite and antigen determinant loci Microbes Infect 2002 4 685 692 10.1016/S1286-4579(02)01587-3 12067827

[B12] Joy DA Feng X Mu J Furuya T Chotivanich K Krettli AU Ho M Wang A White NJ Suh E Beerli P Su XZ Early origin and recent expansion of *Plasmodium falciparum* Science 2003 300 318 321 10.1126/science.1081449 12690197

[B13] Muller I Bockarie M Alpers M Smith T The epidemiology of malaria in Papua New Guinea Trends Parasitol 2003 19 253 259 10.1016/S1471-4922(03)00091-6 12798082

[B14] Cattani JA Tulloch JL Vrbova H Jolley D Gibson FD Moir JS Heywood PF Alpers MP Stevenson A Clancy R The epidemiology of malaria in a population surrounding Madang, Papua New Guinea Am J Trop Med Hyg 1986 35 3 15 3511748 10.4269/ajtmh.1986.35.3

[B15] Bockarie MJ Alexander N Bockarie F Ibam E Barnish G Alpers M The late biting habit of parous Anopheles mosquitoes and pre-bedtime exposure of humans to infective female mosquitoes Trans R Soc Trop Med Hyg 1996 90 23 25 10.1016/S0035-9203(96)90465-4 8730303

[B16] Burkot TR Graves PM Paru R Wirtz RA Heywood PF Human malaria transmission studies in the *Anopheles punctulatus *complex in Papua New Guinea: sporozoite rates, inoculation rates, and sporozoite densities Am J Trop Med Hyg 1988 39 135 144 3044151 10.4269/ajtmh.1988.39.135

[B17] Hii JL Smith T Mai A Ibam E Alpers MP Comparison between anopheline mosquitoes (Diptera: Culicidae) caught using different methods in a malaria endemic area of Papua New Guinea Bull Entomol Res 2000 90 211 219 10.1017/S000748530000033X 10996862

[B18] Hii JL Smith T Vounatsou P Alexander N Mai A Ibam E Alpers MP Area effects of bednet use in a malaria-endemic area in Papua New Guinea Trans R Soc Trop Med Hyg 2001 95 7 13 10.1016/S0035-9203(01)90315-3 11280071

[B19] Kasehagen LJ Mueller I McNamara DT Bockarie MJ Kiniboro B Rare L Lorry K Kastens W Reeder JC Kazura JW Zimmerman PA Changing patterns of Plasmodium blood-stage infections in the Wosera region of Papua New Guinea monitored by light microscopy and high throughput PCR diagnosis Am J Trop Med Hyg 2006 75 588 596 17038678 PMC3728901

[B20] Mueller I Widmer S Michel D Maraga S McNamara DT Kiniboro B Sie A Smith TA Zimmerman PA High sensitivity detection of Plasmodium species reveals positive correlations between infections of different species, shifts in age distribution and reduced local variation in Papua New Guinea Malar J 2009 8 41 55 10.1186/1475-2875-8-41 19284594 PMC2657150

[B21] Paul RE Packer MJ Walmsley M Lagog M Ranford-Cartwright LC Paru R Day KP Mating patterns in malaria parasite populations of Papua New Guinea Science 1995 269 1709 1711 10.1126/science.7569897 7569897

[B22] Sharp PT Highlands malaria: malaria in Enga Province of Papua New Guinea P N G Med J 1982 25 253 260 6764076

[B23] Genton B Al-Yaman F Ginny M Taraika J Alpers MP Relation of anthropometry to malaria morbidity and immunity in Papua New Guinean children Am J Clin Nutr 1998 68 734 741 9734755 10.1093/ajcn/68.3.734

[B24] Gibson RS Heywood A Yaman C Sohlstrom A Thompson LU Heywood P Growth in children from the Wosera subdistrict, Papua New Guinea, in relation to energy and protein intakes and zinc status Am J Clin Nutr 1991 53 782 789 1848037 10.1093/ajcn/53.3.782

[B25] Shankar AH Nutritional modulation of malaria morbidity and mortality J Infect Dis 2000 182 Suppl 1 S37 53 10.1086/315906 10944483

[B26] Shankar AH Genton B Semba RD Baisor M Paino J Tamja S Adiguma T Wu L Rare L Tielsch JM Alpers MP West KP Jr Effect of vitamin A supplementation on morbidity due to *Plasmodium falciparum *in young children in Papua New Guinea: a randomised trial Lancet 1999 354 203 209 10.1016/S0140-6736(98)08293-2 10421302

[B27] Hii JL Smith T Mai A Mellor S Lewis D Alexander N Alpers MP Spatial and temporal variation in abundance of Anopheles (Diptera:Culicidae) in a malaria endemic area in Papua New Guinea J Med Entomol 1997 34 193 205 9103763 10.1093/jmedent/34.2.193

[B28] Burkot TR Dye C Graves PM An analysis of some factors determining the sporozoite rates, human blood indexes, and biting rates of members of the *Anopheles punctulatus *complex in Papua New Guinea Am J Trop Med Hyg 1989 40 229 234 2929848 10.4269/ajtmh.1989.40.229

[B29] Lewis MPe Ethnologue: Languages of the World 2009 16 Dallas: SIL International

[B30] Patel SS King CL Mgone CS Kazura JW Zimmerman PA Glycophorin C (Gerbich antigen blood group) and band 3 polymorphisms in two malaria holoendemic regions of Papua New Guinea Am J Hematol 2004 75 1 5 10.1002/ajh.10448 14695625 PMC3728820

[B31] Cooper RD Waterson DG Frances SP Beebe NW Pluess B Sweeney AW Malaria vectors of Papua New Guinea Int J Parasitol 2009 39 1495 1501 10.1016/j.ijpara.2009.05.009 19505467

[B32] Cooper RD Waterson DG Frances SP Beebe NW Sweeney AW Speciation and distribution of the members of the *Anopheles punctulatus *(Diptera: Culicidae) group in Papua New Guinea J Med Entomol 2002 39 16 27 10.1603/0022-2585-39.1.16 11931251

[B33] Benet A Mai A Bockarie F Lagog M Zimmerman P Alpers MP Reeder JC Bockarie MJ Polymerase chain reaction diagnosis and the changing pattern of vector ecology and malaria transmission dynamics in papua new Guinea Am J Trop Med Hyg 2004 71 277 284 15381806

[B34] Forsyth KP Anders RF Cattani JA Alpers MP Small area variation in prevalence of an S-antigen serotype of *Plasmodium falciparum *in villages of Madang, Papua New Guinea Am J Trop Med Hyg 1989 40 344 350 2653060 10.4269/ajtmh.1989.40.344

[B35] Anderson TJ Su XZ Bockarie M Lagog M Day KP Twelve microsatellite markers for characterization of *Plasmodium falciparum *from finger-prick blood samples Parasitology 1999 119 113 125 10.1017/S0031182099004552 10466118

[B36] Falk N Maire N Sama W Owusu-Agyei S Smith T Beck HP Felger I Comparison of PCR-RFLP and Genescan-based genotyping for analyzing infection dynamics of *Plasmodium falciparum* Am J Trop Med Hyg 2006 74 944 950 16760501

[B37] Glaubitz JC CONVERT: A user-friendly program to reformat diploid genotypic data for commonly used population genetic software packages Molecular Ecology Notes 2004 4 309 310 10.1111/j.1471-8286.2004.00597.x

[B38] Excoffier L Laval G Schneider S Arlequin ver. 3.0: An integrated software package for population genetics data analysis Evolutionary Bioinformatics Online 2005 1 47 50 19325852 PMC2658868

[B39] Hurlbert SH The non-concept of species diversity: a critique and alternative parameters Ecology 1971 52 577 586 10.2307/1934145 28973811

[B40] Goudet J FSTAT (Version 1.2): A computer program to calculate F-statistics Journal of Heredity 1995 86 485 486

[B41] Haubold B Hudson RR LIAN 3.0: detecting linkage disequilibrium in multilocus data. Linkage Analysis Bioinformatics 2000 16 847 848 10.1093/bioinformatics/16.9.847 11108709

[B42] Michalakis Y Excoffier L A generic estimation of population subdivision using distances between alleles with special reference for microsatellite loci Genetics 1996 142 1061 1064 8849912 10.1093/genetics/142.3.1061 PMC1207006

[B43] Slatkin M A measure of population subdivision based on microsatellite allele frequencies Genetics 1995 139 457 462 7705646 10.1093/genetics/139.1.457 PMC1206343

[B44] Anderson TJ Su XZ Roddam A Day KP Complex mutations in a high proportion of microsatellite loci from the protozoan parasite *Plasmodium falciparum* Mol Ecol 2000 9 1599 1608 10.1046/j.1365-294x.2000.01057.x 11050555

[B45] Manly BJF Randomisation and Monte Carlo methods in biology 1991 London: Chapman and Hall

[B46] Pritchard JK Stephens M Donnelly P Inference of population structure using multilocus genotype data 2000 155 945 10835412 10.1093/genetics/155.2.945 PMC1461096

[B47] Pritchard JK Wen X Falush D Documentation for *structure *software: Version 2.2 2007 http://pritch.bsd.uchicago.edu/structure.html

[B48] Evanno G Regnaut S Goudet J Detecting the number of clusters of individuals using the software STRUCTURE: a simulation study Mol Ecol 2005 14 2611 2620 10.1111/j.1365-294X.2005.02553.x 15969739

[B49] Shannon P Markiel A Ozier O Baliga NS Wang JT Ramage D Amin N Schwikowski B Ideker T Cytoscape: a software environment for integrated models of biomolecular interaction networks Genome Res 2003 13 2498 2504 10.1101/gr.1239303 14597658 PMC403769

[B50] Kamada TaK S A simple method for computing general position in displaying three-dimensional objects Computer Vision, Graphics and Image Processing 1988 41 43 56 10.1016/0734-189X(88)90116-8

[B51] Smith JM Smith NH O'Rourke M Spratt BG How clonal are bacteria? Proc Natl Acad Sci USA 1993 90 4384 4388 10.1073/pnas.90.10.4384 8506277 PMC46515

[B52] Li CC Population Genetics 1955 Chicago: University of Chicago Press

[B53] Annan Z Durand P Ayala FJ Arnathau C Awono-Ambene P Simard F Razakandrainibe FG Koella JC Fontenille D Renaud F Population genetic structure of *Plasmodium falciparum *in the two main African vectors, *Anopheles gambiae *and *Anopheles funestus* Proc Natl Acad Sci USA 2007 104 7987 7992 10.1073/pnas.0702715104 17470800 PMC1876559

