# Supplementary material for: Multilocus haplotypes reveal variable levels of diversity and population structure of Plasmodium falciparum in Papua New Guinea, a region of intense perennial transmission
Source: Malar J. 2010 Nov 23;9:336. doi: 10.1186/1475-2875-9-336 (PMC3002378; doi:10.1186/1475-2875-9-336)
Supplement: Additional file 1 — Intensity of P. falciparum transmission in Papua New Guinea. Two correlates of parasite transmission, the infection prevalence and the proportion of infected people carrying multiple P. falciparum clones, were estimated by msp2 genotyping. [file 1475-2875-9-336-S1.PDF]

Additional file 1. Intensity of *P. falciparum* transmission in Papua New Guinea. Two correlates of parasite transmission, the infection prevalence and the proportion of infected people carrying multiple *P. falciparum* clones, were estimated by *msp2* genotyping.

Population	Number of samples	Infection prevalence (%)	Proportion of infections with multiple clones (%)
Utu	***395***	***40***	***45***
Utu	395	39	45
Malala	***387***	***33***	***39***
Amiten/Susure	172	33	53
Malala/Suraten	65	34	23
Wakorma	150	37	31
Mugil	***493***	***44***	***44***
Dimer	74	43	44
Karkum	209	42	42
Matukar/Bunu	210	47	46
Wosera	***872***	***28***	***45***
Gwinyingi	119	38	40
Patigo	113	12	0
Nindigo	216	44	45
Kitikum	124	39	65
Wisokum	200	17	47
Tatemba	100	25	44
**TOTAL**	**2147**	**35**	**44**
