# Supplementary material for: Multilocus haplotypes reveal variable levels of diversity and population structure of Plasmodium falciparum in Papua New Guinea, a region of intense perennial transmission
Source: Malar J. 2010 Nov 23;9:336. doi: 10.1186/1475-2875-9-336 (PMC3002378; doi:10.1186/1475-2875-9-336)
Supplement: Additional file 2 — Associations between transmission intensity and genetic diversity in Papua New Guinea. Matrix of Spearmans rank correlation coefficients (ρ) and in brackets, associated p-values. [file 1475-2875-9-336-S2.PDF]

Additional file 2. Associations between transmission intensity and genetic diversity in Papua New Guinea. Matrix of Spearmans rank correlation coefficients (ρ) and in brackets, associated p-values.

	**Infection prevalence (%)**	**Proportion of infections containing multiple clones (%)**
Village		
R_s	0.30 (0.29)	0.15 (0.60)
H_e	0.24 (0.40)	0.19 (0.48)
Catchment[a]		
R_s	-0.40	0.11
H_e	0	- 0.95

[a]p-value not calculated due to the small number of pairwise comparisons
