# Supplementary material for: Multilocus haplotypes reveal variable levels of diversity and population structure of Plasmodium falciparum in Papua New Guinea, a region of intense perennial transmission
Source: Malar J. 2010 Nov 23;9:336. doi: 10.1186/1475-2875-9-336 (PMC3002378; doi:10.1186/1475-2875-9-336)
Supplement: Additional file 3 — Microsatellite allele frequencies for Plasmodium falciparum populations from Papua New Guinea. n/a. [file 1475-2875-9-336-S3.PDF]

**Additional file 3.** Microsatellite allele frequencies for *Plasmodium falciparum* populations from Papua New Guinea

| Locus | Allele | No. of Repeats | WOSERA | | | | | | UTU | MALALA | | | MUGIL | | | Overall | Private alleles |
|---|---|---|---|---|---|---|---|---|---|---|---|---|---|---|---|---|---|
| | | | Gwiyning | Patigo | Nindigo | Tatemba | Wisokum | Kitikum | Utu | Amiten/ Susure | Malala/ Suraten | Wakorma | Dimer | Karkum | Matukar/ Bunu | | |
| **TA1** | 1 | 14 | 0.79 | 0.80 | 0.65 | | 0.85 | 0.64 | | | | | 0.36 | 0.20 | 0.17 | 0.26 | |
| | 2 | 15 | | 0.10 | | | | | | | | | | | | 0.00 | Patigo |
| | 3 | 17 | | | | | | | | | | | | 0.07 | | 0.00 | Karkum |
| | 4 | 18 | | | | | | | | 0.10 | | | | | | 0.01 | Amiten/Susure |
| | 5 | 19 | | | | | | | | 0.38 | 0.67 | 0.52 | | | | 0.13 | |
| | 6 | 20 | 0.05 | | 0.12 | | | | | | | | | | 0.08 | 0.02 | |
| | 7 | 21 | | | 0.12 | | | | 0.06 | | | | | | 0.08 | 0.03 | |
| | 8 | 22 | | | 0.06 | 0.10 | | 0.09 | 0.12 | 0.05 | | | 0.09 | 0.27 | 0.17 | 0.07 | |
| | 9 | 23 | 0.05 | | | 0.20 | 0.08 | | 0.17 | 0.10 | 0.11 | 0.09 | 0.45 | 0.20 | 0.33 | 0.13 | |
| | 10 | 24 | 0.11 | | | 0.20 | | 0.27 | 0.33 | 0.14 | 0.22 | 0.21 | 0.09 | | | 0.16 | |
| | 11 | 25 | | 0.10 | 0.06 | 0.10 | 0.08 | | 0.06 | | | | | 0.20 | 0.17 | 0.05 | |
| | 12 | 26 | | | | 0.10 | | | 0.12 | 0.10 | | 0.06 | | | | 0.05 | |
| | 13 | 27 | | | | | | | 0.08 | 0.05 | | 0.03 | | 0.07 | | 0.03 | |
| | 14 | 28 | | | | 0.30 | | | 0.04 | 0.05 | | 0.06 | | | | 0.03 | |
| | 15 | 29 | | | | | | | | | | 0.03 | | | | 0.00 | Wakorma |
| | 16 | 30 | | | | | | | 0.02 | | | | | | | 0.00 | Utu |
| | 17 | 33 | | | | | | | | 0.05 | | | | | | 0.00 | Amiten/Susure |
| | 18 | 36 | | | | | | | 0.02 | | | | | | | 0.00 | Utu |
| | *Samples* | | *19* | *10* | *17* | *10* | *13* | *11* | *52* | *21* | *9* | *33* | *11* | *15* | *12* | *233* | |
| ***TAA60*** | 1 | 4 | | | | | | | | 0.05 | | | | | | 0.00 | Amiten/Susure |
| | 2 | 5 | | | | | | | 0.03 | | | | 0.13 | 0.04 | | 0.02 | |
| | 3 | 6 | | | | | 0.10 | 0.10 | | 0.05 | | | 0.13 | | | 0.02 | |
| | 4 | 7 | 0.17 | 0.10 | 0.27 | 0.10 | | 0.20 | 0.19 | 0.19 | 0.25 | 0.26 | 0.06 | 0.27 | 0.43 | 0.21 | |
| | 5 | 8 | 0.44 | 0.10 | 0.24 | 0.30 | 0.40 | 0.40 | 0.61 | 0.38 | 0.33 | 0.29 | 0.44 | 0.46 | 0.43 | 0.41 | |
| | 6 | 9 | | | 0.03 | 0.20 | | | 0.06 | 0.19 | | 0.10 | 0.06 | 0.19 | 0.10 | 0.08 | |
| | 7 | 10 | | | 0.03 | 0.10 | 0.20 | 0.10 | 0.03 | 0.05 | 0.17 | 0.03 | 0.06 | | | 0.04 | |
| | 8 | 11 | 0.11 | 0.10 | 0.03 | 0.20 | 0.30 | 0.20 | 0.02 | | | 0.06 | | | | 0.05 | |
| | 9 | 12 | | | | | | | 0.02 | | | | | 0.04 | | 0.01 | |
| | 10 | 13 | 0.28 | 0.70 | 0.39 | | | | 0.05 | | | | | | | 0.10 | |
| | 11 | 17 | | | | 0.10 | | | | 0.10 | 0.25 | 0.26 | 0.13 | | 0.05 | 0.06 | |
| | *Samples* | | *18* | *10* | *33* | *10* | *10* | *10* | *64* | *21* | *12* | *31* | *16* | *26* | *21* | *282* | |
| | 1 | 19 | | | | | | | | 0.05 | | | | | | 0.00 | Amiten/Susure |
| | 2 | 22 | | | 0.04 | | | 0.10 | | | 0.14 | 0.04 | | | | 0.02 | |
| | 3 | 23 | 0.07 | | 0.15 | 0.09 | 0.25 | 0.30 | 0.08 | 0.23 | 0.14 | 0.19 | 0.17 | 0.09 | 0.36 | 0.16 | |

| | | | | | | | | | | | | | | | | | |
|---|---|---|---|---|---|---|---|---|---|---|---|---|---|---|---|---|---|
| | 4 | 24 | | 0.17 | | | | | | | | | 0.08 | | | 0.01 | |
| | 5 | 25 | 0.07 | | 0.22 | | 0.08 | | 0.14 | 0.14 | 0.14 | 0.04 | | 0.09 | 0.05 | 0.10 | |
| | 6 | 26 | 0.33 | 0.50 | 0.22 | 0.36 | 0.33 | | 0.14 | 0.09 | | 0.08 | | 0.05 | 0.23 | 0.16 | |
| | 7 | 27 | 0.07 | 0.17 | 0.15 | 0.27 | 0.17 | 0.10 | 0.05 | 0.05 | | 0.04 | | 0.14 | 0.05 | 0.08 | |
| | 8 | 28 | | | 0.04 | | | | | | | | | | 0.05 | 0.01 | |
| | 9 | 29 | | | | 0.09 | | | 0.05 | 0.05 | | 0.08 | 0.08 | 0.09 | | 0.04 | |
| ***Polyalpha*** | 10 | 30 | 0.20 | | | 0.09 | 0.08 | 0.10 | 0.05 | 0.05 | 0.14 | 0.08 | 0.17 | | 0.09 | 0.07 | |
| | 11 | 31 | | | 0.04 | | | | 0.02 | 0.05 | | 0.04 | | | 0.09 | 0.02 | |
| | 12 | 32 | 0.07 | | 0.07 | | | 0.30 | 0.17 | 0.05 | 0.14 | 0.19 | 0.08 | 0.18 | | 0.11 | |
| | 13 | 33 | 0.13 | | 0.07 | 0.09 | | | 0.02 | 0.23 | 0.29 | 0.15 | 0.17 | 0.18 | | 0.09 | |
| | 14 | 34 | | | | | | | | 0.05 | | 0.04 | 0.08 | | 0.09 | 0.02 | |
| | 15 | 35 | 0.07 | | | | 0.08 | | 0.03 | | | | 0.17 | 0.09 | | 0.03 | |
| | 16 | 36 | | 0.17 | | | | | 0.27 | | | | | 0.09 | | 0.08 | |
| | 17 | 37 | | | | | | | | | | 0.04 | | | | 0.00 | Wakorma |
| | 18 | 43 | | | | | | 0.10 | | | | | | | | 0.00 | Kitikum |
| | *Samples* | | *15* | *6* | *27* | *11* | *12* | *10* | *64* | *22* | *7* | *26* | *12* | *22* | *22* | *256* | |
| | 1 | 3 | | 0.15 | | | | | | | | | | | | 0.01 | Patigo |
| | 2 | 4 | | 0.08 | | | | | | | | | | 0.04 | 0.04 | 0.01 | |
| | 3 | 5 | 0.14 | 0.08 | 0.06 | | | | | | | | 0.07 | 0.15 | | 0.04 | |
| | 4 | 6 | | | | | | | | | | | | 0.04 | | 0.00 | Karkum |
| | 5 | 7 | 0.05 | | 0.09 | | | 0.15 | 0.02 | | 0.36 | 0.26 | | 0.04 | 0.04 | 0.07 | |
| | 6 | 8 | 0.18 | 0.15 | 0.34 | 0.30 | 0.46 | 0.08 | 0.42 | 0.09 | 0.18 | 0.19 | 0.29 | | 0.12 | 0.24 | |
| | 7 | 9 | 0.09 | 0.31 | 0.22 | 0.20 | 0.08 | 0.23 | 0.22 | | | | 0.21 | 0.19 | 0.24 | 0.16 | |
| ***ARA2*** | 8 | 10 | 0.27 | | 0.13 | 0.10 | 0.23 | 0.08 | 0.12 | 0.18 | | 0.30 | | 0.11 | 0.16 | 0.14 | |
| | 9 | 11 | 0.09 | 0.15 | 0.06 | 0.20 | 0.15 | 0.23 | 0.13 | 0.55 | 0.45 | 0.22 | 0.43 | 0.33 | 0.32 | 0.23 | |
| | 10 | 12 | 0.09 | 0.08 | 0.03 | 0.10 | 0.08 | 0.08 | 0.08 | 0.14 | | | | 0.07 | 0.04 | 0.06 | |
| | 11 | 13 | 0.00 | | 0.06 | 0.10 | | 0.15 | | 0.05 | | 0.04 | | 0.04 | 0.04 | 0.03 | |
| | 12 | 14 | 0.05 | | | | | | 0.02 | | | | | | | 0.01 | |
| | 13 | 16 | 0.05 | | | | | | | | | | | | | 0.00 | Gwinyingi |
| | *Samples* | | *22* | *13* | *32* | *10* | *13* | *13* | *60* | *22* | *11* | *27* | *14* | *27* | *25* | *289* | |
| | 1 | 5 | 0.05 | | 0.03 | 0.20 | | | | | | | | | | 0.01 | |
| | 2 | 6 | 0.15 | 0.17 | 0.57 | 0.40 | 0.31 | 0.54 | 0.48 | 0.37 | 0.20 | 0.17 | 0.29 | 0.08 | 0.17 | 0.33 | |
| | 3 | 7 | 0.30 | 0.42 | 0.23 | 0.30 | 0.46 | 0.08 | 0.24 | 0.26 | 0.40 | 0.29 | 0.43 | 0.25 | 0.42 | 0.29 | |
| | 4 | 8 | 0.35 | 0.17 | 0.07 | 0.10 | 0.15 | 0.23 | 0.11 | 0.05 | | 0.04 | 0.07 | 0.29 | 0.08 | 0.13 | |
| | 5 | 9 | 0.15 | 0.25 | | | 0.08 | 0.08 | 0.06 | 0.32 | 0.40 | 0.46 | 0.07 | 0.25 | 0.17 | 0.16 | |
| ***Pfg377*** | 6 | 10 | | | 0.07 | | | | | | | | 0.14 | 0.08 | 0.00 | 0.02 | |
| | 7 | 12 | | | | | | 0.08 | 0.10 | | | | | | 0.08 | 0.03 | |
| | 8 | 13 | | | 0.03 | | | | | | | | | | | 0.00 | Nindigo |
| | 9 | 17 | | | | | | | | | | 0.04 | | 0.04 | 0.04 | 0.01 | |
| | 10 | 18 | | | | | | | | | | | | | 0.04 | 0.00 | Matukar/Bunu |
| | *Samples* | | *20* | *12* | *30* | *10* | *13* | *13* | *62* | *19* | *10* | *24* | *14* | *24* | *24* | *275* | |

| | | | | | | | | | | | | | | | | | |
|---|---|---|---|---|---|---|---|---|---|---|---|---|---|---|---|---|---|
| | 1 | 9 | | | | | | | | | | 0.04 | | | | 0.00 | Wakorma |
| | 2 | 10 | 0.17 | 0.22 | 0.09 | | 0.08 | | 0.03 | | 0.20 | 0.25 | 0.06 | | 0.04 | 0.08 | |
| | 3 | 12 | | | | | | | 0.02 | | | | | | | 0.00 | Utu |
| | 4 | 13 | | | | | | | | | | | 0.06 | | | 0.00 | Dimer |
| | 5 | 15 | | 0.11 | 0.03 | | 0.08 | 0.30 | | 0.15 | 0.20 | 0.11 | 0.06 | | | 0.05 | |
| | 6 | 16 | 0.06 | 0.11 | 0.03 | | | | 0.02 | 0.10 | 0.10 | 0.04 | | | 0.08 | 0.04 | |
| ***TAA87*** | 7 | 17 | 0.11 | 0.33 | 0.06 | | 0.08 | 0.10 | 0.08 | 0.30 | 0.40 | 0.43 | 0.19 | 0.48 | 0.40 | 0.22 | |
| | 8 | 18 | 0.22 | | 0.29 | 0.36 | 0.42 | 0.30 | 0.27 | 0.30 | 0.10 | | 0.38 | 0.36 | 0.20 | 0.25 | |
| | 9 | 19 | 0.22 | 0.22 | 0.15 | 0.09 | 0.17 | | 0.19 | | | 0.11 | | 0.04 | 0.08 | 0.11 | |
| | 10 | 20 | 0.17 | | 0.26 | 0.55 | 0.17 | 0.20 | 0.35 | | | 0.04 | 0.19 | 0.12 | 0.20 | 0.20 | |
| | 11 | 21 | | | 0.09 | | | 0.10 | | 0.10 | | | | | | 0.02 | |
| | 12 | 22 | 0.06 | | | | | | 0.03 | 0.05 | | | 0.06 | | | 0.02 | |
| | *Samples* | | *18* | *9* | *34* | *11* | *12* | *10* | *62* | *20* | *10* | *28* | *16* | *25* | *25* | *280* | |
| | 1 | 7 | 0.06 | | | | 0.11 | 0.22 | | 0.06 | 0.18 | 0.07 | | | 0.11 | 0.04 | |
| | 2 | 8 | 0.75 | 0.40 | 0.45 | 0.20 | 0.67 | 0.33 | 0.82 | 0.53 | 0.18 | 0.17 | 0.47 | 0.47 | 0.58 | 0.53 | |
| | 3 | 9 | 0.06 | 0.20 | 0.30 | 0.50 | 0.11 | 0.11 | 0.02 | 0.18 | 0.09 | 0.21 | 0.20 | 0.16 | 0.05 | 0.14 | |
| | 4 | 10 | | | | | 0.11 | | | | | | | 0.05 | | 0.01 | |
| | 5 | 11 | | | 0.10 | | | | 0.02 | 0.06 | 0.09 | | | | | 0.02 | |
| | 6 | 12 | 0.06 | 0.10 | | | | | | 0.12 | 0.18 | 0.21 | | 0.05 | 0.11 | 0.06 | |
| | 7 | 13 | | | | | | 0.22 | 0.02 | | 0.09 | | | | | 0.02 | |
| | 8 | 14 | | | 0.05 | | | | 0.05 | | | | | | | 0.02 | |
| | 9 | 15 | | | | | | | | | | | 0.13 | 0.05 | 0.05 | 0.02 | |
| ***TAA42*** | 10 | 23 | | | | | | | 0.03 | | | | | | | 0.01 | Utu |
| | 11 | 27 | 0.06 | | | 0.10 | | | | | | | 0.07 | | | 0.01 | |
| | 12 | 28 | | | 0.05 | 0.10 | | | | | | 0.07 | 0.07 | 0.11 | 0.05 | 0.03 | |
| | 13 | 29 | | | | | | | 0.03 | | | 0.03 | | 0.05 | | 0.02 | |
| | 14 | 30 | | | | 0.10 | | | | 0.06 | | | | 0.05 | | 0.01 | |
| | 15 | 33 | | | | | | | 0.02 | | | | | | | 0.00 | Utu |
| | 16 | 40 | | | | | | 0.11 | | | | 0.07 | | | | 0.01 | |
| | 17 | 41 | | 0.10 | | | | | | | | | | | | 0.00 | Patigo |
| | 18 | 42 | | 0.20 | 0.05 | | | | | | 0.18 | 0.17 | 0.07 | | 0.05 | 0.05 | |
| | *Samples* | | *16* | *10* | *20* | *10* | *9* | *9* | *62* | *17* | *11* | *29* | *15* | *19* | *19* | *246* | |
| | 1 | 1 | | | | | | | | 0.05 | | | | | | 0.00 | Amiten/Susure |
| | 2 | 3 | 0.05 | 0.17 | | | | | | | | | | | | 0.01 | |
| | 3 | 5 | | | | | | | | | 0.10 | | | | | 0.00 | Malala/Suraten |
| | 4 | 7 | | | | | | | 0.02 | 0.05 | | 0.03 | | 0.05 | 0.05 | 0.02 | |
| | 5 | 8 | | | 0.09 | | 0.08 | | 0.41 | 0.21 | 0.20 | 0.06 | 0.42 | 0.45 | 0.27 | 0.20 | |
| | 6 | 9 | 0.10 | | | | | 0.08 | 0.41 | 0.32 | 0.10 | 0.25 | 0.17 | 0.25 | 0.27 | 0.20 | |
| | 7 | 10 | 0.29 | 0.17 | 0.29 | 0.27 | 0.08 | 0.15 | 0.08 | 0.21 | 0.10 | 0.16 | 0.00 | 0.10 | 0.05 | 0.15 | |
| | 8 | 11 | 0.29 | 0.25 | 0.54 | 0.36 | 0.33 | 0.46 | 0.03 | 0.11 | 0.10 | 0.19 | 0.17 | 0.05 | 0.05 | 0.21 | |
| | 9 | 12 | 0.10 | 0.08 | 0.03 | 0.09 | 0.08 | 0.08 | 0.02 | 0.05 | 0.40 | 0.13 | | 0.05 | 0.18 | 0.08 | |

| | | | | | | | | | | | | | | | | | |
|---|---|---|---|---|---|---|---|---|---|---|---|---|---|---|---|---|---|
| *PfPK2* | 10 | 13 | | | | 0.09 | | | | | | | | | | 0.00 | Tatemba |
| | 11 | 14 | 0.05 | | 0.06 | 0.09 | 0.08 | 0.08 | | | | | | | | 0.02 | |
| | 12 | 15 | | 0.17 | | 0.09 | | | | | | 0.03 | | | | 0.01 | |
| | 13 | 16 | | | | | 0.08 | 0.08 | 0.02 | | | 0.03 | | | | 0.01 | |
| | 14 | 17 | | | | | 0.08 | | | | | 0.06 | 0.17 | | | 0.02 | |
| | 15 | 18 | 0.05 | | | | 0.17 | | | | | | | | 0.05 | 0.01 | |
| | 16 | 19 | | | | | | | | | | | | 0.05 | | 0.00 | Karkum |
| | 17 | 20 | | 0.08 | | | | | | | | 0.06 | | | | 0.01 | |
| | 18 | 21 | | | | | | 0.08 | 0.02 | | | | 0.08 | | 0.09 | 0.02 | |
| | 19 | 22 | 0.10 | 0.08 | | | | | | | | | | | | 0.01 | |
| | *Samples* | | *21* | *12* | *35* | *11* | *12* | *13* | *59* | *19* | *10* | *32* | *12* | *20* | *22* | *278* | |
| *TAA81* | 1 | 6 | 0.24 | | 0.03 | 0.25 | | 0.33 | 0.05 | 0.18 | | 0.06 | 0.07 | 0.00 | 0.04 | 0.07 | |
| | 2 | 7 | 0.24 | 0.38 | 0.32 | 0.13 | 0.33 | 0.17 | 0.24 | 0.14 | | 0.06 | | 0.11 | 0.04 | 0.17 | |
| | 3 | 8 | 0.10 | 0.15 | 0.29 | 0.13 | 0.22 | | 0.12 | 0.09 | 0.17 | 0.24 | 0.20 | 0.14 | 0.11 | 0.16 | |
| | 4 | 9 | | | | 0.13 | | | 0.05 | 0.14 | 0.17 | 0.06 | 0.07 | 0.07 | 0.15 | 0.06 | |
| | 5 | 10 | 0.14 | 0.15 | 0.06 | 0.13 | 0.33 | 0.17 | 0.08 | 0.14 | 0.00 | 0.09 | | 0.11 | 0.15 | 0.10 | |
| | 6 | 11 | 0.29 | 0.31 | 0.19 | 0.25 | | 0.17 | 0.42 | 0.27 | 0.67 | 0.41 | 0.33 | 0.21 | 0.19 | 0.31 | |
| | 7 | 12 | | | | | | | 0.02 | | | 0.03 | 0.33 | 0.32 | 0.26 | 0.08 | |
| | 8 | 13 | | | 0.03 | | 0.11 | 0.17 | | 0.05 | | 0.03 | | 0.04 | 0.04 | 0.02 | |
| | 9 | 14 | | | | | | | 0.03 | | | | | | | 0.01 | Utu |
| | 10 | 17 | | | | | | | | | | | | | 0.04 | 0.00 | Matukar/Bunu |
| | 11 | 20 | | | 0.06 | | | | | | | 0.03 | | | | 0.01 | |
| | *Samples* | | *21* | *13* | *31* | *8* | *9* | *6* | *66* | *22* | *12* | *34* | *15* | *28* | *27* | *292* | |
| *2490* | 1 | 6 | 0.06 | | | | | | | 0.05 | | | | 0.04 | | 0.01 | |
| | 2 | 7 | | | | | 0.17 | | | 0.05 | | | | 0.07 | | 0.01 | |
| | 3 | 8 | | | 0.03 | | | | | | 0.08 | 0.03 | | | | 0.01 | |
| | 4 | 9 | 0.78 | 0.83 | 0.74 | 0.71 | 0.83 | 0.50 | 0.88 | 0.55 | 0.33 | 0.50 | 0.33 | 0.07 | 0.33 | 0.59 | |
| | 5 | 10 | 0.11 | 0.08 | 0.20 | 0.14 | | 0.25 | 0.09 | 0.05 | 0.17 | 0.09 | 0.53 | 0.79 | 0.48 | 0.24 | |
| | 6 | 11 | | 0.08 | | 0.14 | | | | 0.05 | | 0.06 | | | 0.07 | 0.02 | |
| | 7 | 12 | | | 0.03 | | | | | 0.05 | | 0.09 | 0.13 | 0.04 | 0.07 | 0.03 | |
| | 8 | 13 | | | | | | | | 0.23 | 0.17 | 0.21 | | | | 0.05 | |
| | 9 | 14 | 0.06 | | | | | | 0.03 | | 0.25 | 0.03 | | | 0.04 | 0.03 | |
| | 10 | 15 | | | | | | 0.25 | | | 0.00 | | | | | 0.01 | Kitikum |
| | *Samples* | | *18* | *12* | *35* | *7* | *6* | *8* | *64* | *22* | *12* | *34* | *15* | *28* | *27* | *288* | |
