# Supplementary material for: Multilocus haplotypes reveal variable levels of diversity and population structure of Plasmodium falciparum in Papua New Guinea, a region of intense perennial transmission
Source: Malar J. 2010 Nov 23;9:336. doi: 10.1186/1475-2875-9-336 (PMC3002378; doi:10.1186/1475-2875-9-336)
Supplement: Additional file 4 — Genetic differentiation between Plasmodium falciparum populations of Papua New Guinea. Matrix of pairwise FST (lower diagonal) and RST values (upper diagonal) between villages. [file 1475-2875-9-336-S4.PDF]

Additional file 4. Genetic differentiation between *Plasmodium falciparum* populations of Papua New Guinea

Matrix of pairwise F_{ST} (lower diagonal) and R_{ST} values (upper diagonal) between villages

	Wosera						Utu	Malala			Mugil		
	Gwinyingi	Patigo#	Nindigo	Tatemba#	Wisokum#	Kitikum#	Utu	Amiten/ Susure	Malala/ Suraten#	Wakorma	Dimer#	Karkum	Matukar/ Bunu
Gwinyingi		0.05	0.03	0.06	0	0.02	0.06**	0.05*	0.11**	0.06**	0.08**	0.13**	0.08**
Patigo#	0.05		0.10*	0.21*	0.07	0.17*	0.11**	0.08*	0.13**	0.07**	0.12**	0.17**	0.11**
Nindigo	0.05**	0.05		0.05	0.07	0.04	0.09**	0.09**	0.15**	0.09**	0.10**	0.17**	0.11**
Tatemba#	0.05	0.07	0.02		0.12	0.07	0.12**	0.07*	0.11**	0.08**	0.07	0.13**	0.08*
Wisokum#	0.02	0.06	0.04	0.03		0	0.06**	0.03	0.09**	0.05*	0.04	0.12**	0.05
Kitikum#	0.06	0.10*	0.04	0.05	0.06		0.10**	0.02	0.07	0.04	0.04	0.09**	0.05
Utu	0.17**	0.33**	0.13**	0.20**	0.25**	0.20**		0.07*	0.27**	0.28**	0.11*	0.05*	0.14**
Amiten/ Susure	0.10*	0.25**	0.14**	0.17**	0.19**	0.11*	0.10**		0.16**	0.17**	0.09	0.10**	0.09*
Malala/ Suraten#	0.06	0.02	0.14*	0.26**	0.12	0.09	0.15**	0.04		0	0.09	0.18**	0.08
Wakorma	0.04	0.01	0.12**	0.19**	0.05	0.05	0.13**	0.03	0.02		0.08	0.15**	0.07*
Dimer#	0.01	0.07	0.07	0.12	0	0	0.10**	0.05*	0.08*	0.08**		0	0
Karkum	0.13*	0.24**	0.14**	0.27**	0.22**	0.16*	0.17**	0.10**	0.12**	0.11**	0.04		0.01
Matukar/ Bunu	0.08*	0.17*	0.11**	0.17*	0.11*	0.07	0.11**	0.05*	0.07*	0.06**	0.02	0.04*	

small sample size (n ≤ 14); ** $P < 0.01$; *$P < 0.05$; all negative values were converted to 0
