# Supplementary material for: Multilocus haplotypes reveal variable levels of diversity and population structure of Plasmodium falciparum in Papua New Guinea, a region of intense perennial transmission
Source: Malar J. 2010 Nov 23;9:336. doi: 10.1186/1475-2875-9-336 (PMC3002378; doi:10.1186/1475-2875-9-336)
Supplement: Additional file 5 — Associations between geographic distance (km) and pairwise genetic differentiation in Papua New Guinea. Matrix of Mantel correlation results and in brackets, p-values. [file 1475-2875-9-336-S5.PDF]

Additional file 5. Associations between geographic distance (km) and pairwise genetic differentiation in Papua New Guinea. Matrix of Mantel correlation results and in brackets, p-values.

	**Distance (km)**	
	Village	*Catchment*[a]
F_{ST}	0.19 (0.41)	-0.45
R_{ST}	0.16 (0.49)	0.000779

[a]p-values not calculated due to the small number of pairwise comparisons
