# Supplementary material for: Multilocus haplotypes reveal variable levels of diversity and population structure of Plasmodium falciparum in Papua New Guinea, a region of intense perennial transmission
Source: Malar J. 2010 Nov 23;9:336. doi: 10.1186/1475-2875-9-336 (PMC3002378; doi:10.1186/1475-2875-9-336)

Additional file 6. Definition of the most probable number of clusters for 318 *Plasmodium falciparum* microsatellite haplotypes from Papua New Guinea

Structure analysis (ΔK plots) for A) all haplotypes (B) inland and coastal datasets and (C) and each of the catchments.

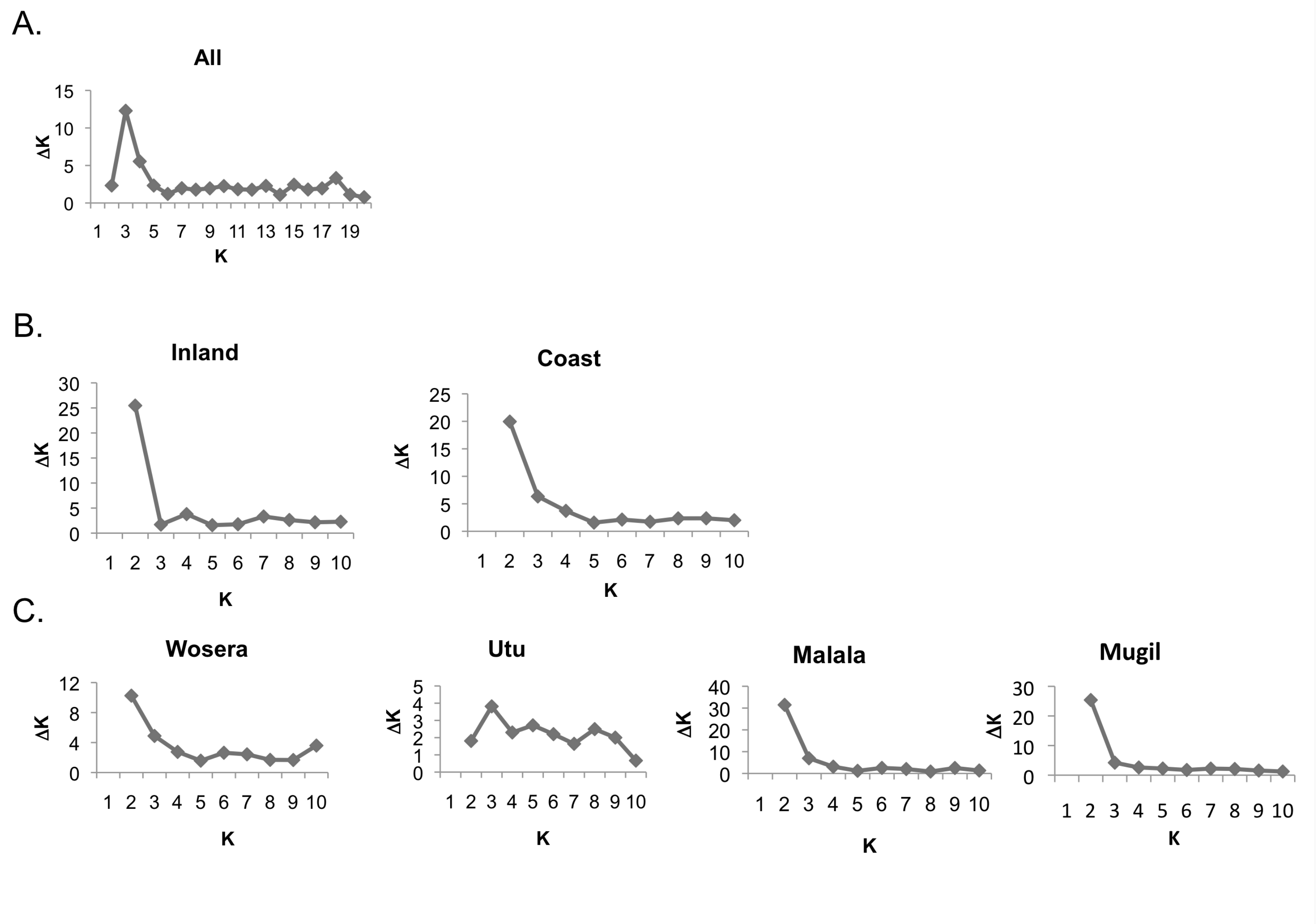

Supplement: Additional file 6 — Definition of the most probable number of clusters for 318 Plasmodium falciparum microsatellite haplotypes from Papua New Guinea. Structure analysis (ΔK plots) for A) all haplotypes (B) inland and coastal datasets and (C) and each of the catchments. [file 1475-2875-9-336-S6.PDF]
