# Supplementary material for: Multilocus haplotypes reveal variable levels of diversity and population structure of Plasmodium falciparum in Papua New Guinea, a region of intense perennial transmission
Source: Malar J. 2010 Nov 23;9:336. doi: 10.1186/1475-2875-9-336 (PMC3002378; doi:10.1186/1475-2875-9-336)

Additional file 7. Network analysis of 318 *Plasmodium falciparum* haplotypes within four catchment areas of Papua New Guinea. Weighted network of haplotypes showing relationships among individuals for each catchment. (A) Wosera (B) Utu (C) Malala and (D) Mugil. Each coloured circle represents a haplotype (node), and black lines indicate shared alleles among individual haplotypes. The threshold for a connection was set at three matching loci between two haplotypes.

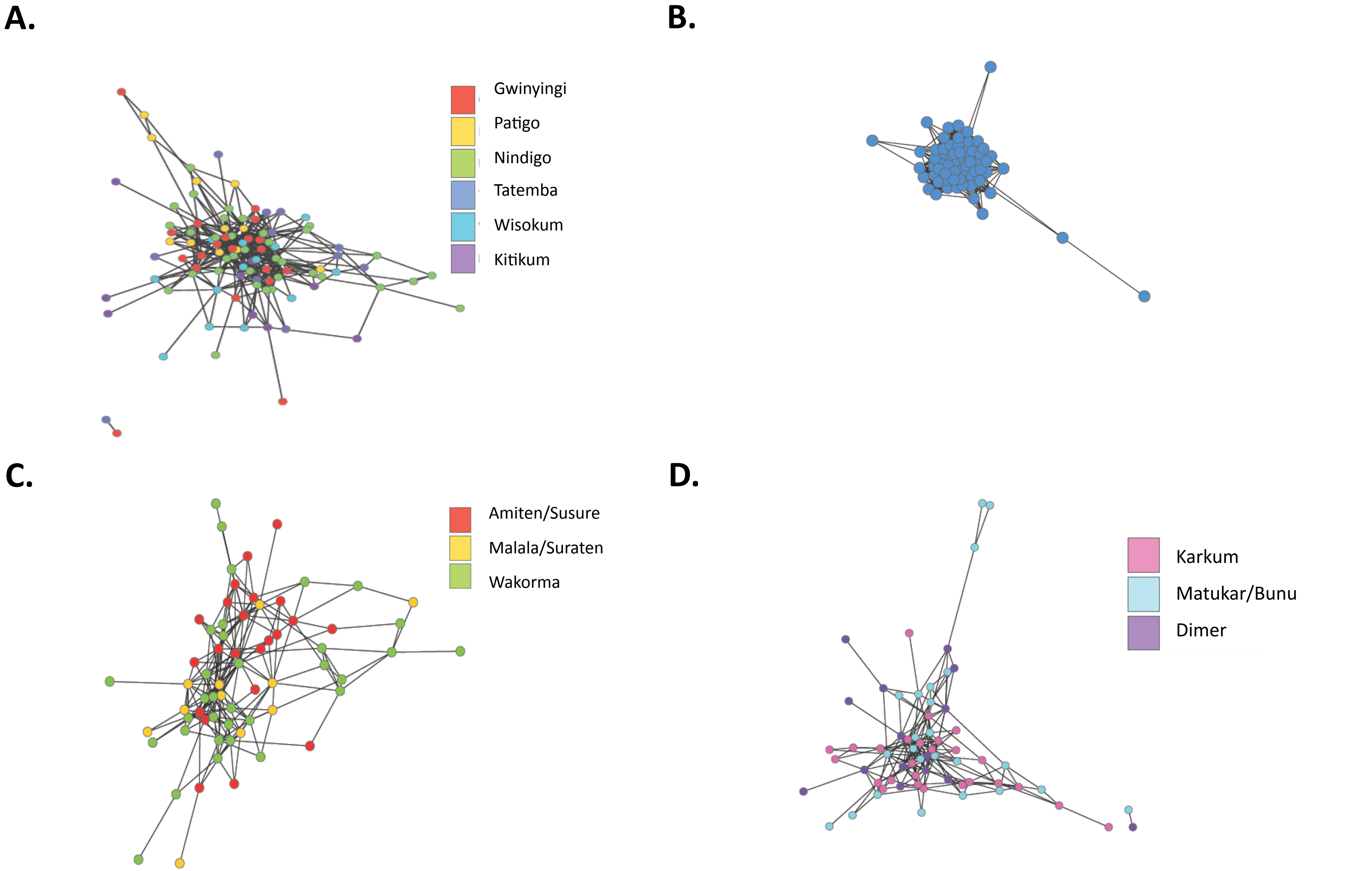

Supplement: Additional file 7 — Network analysis of 318 Plasmodium falciparum haplotypes within four catchment areas of Papua New Guinea. Weighted network of haplotypes showing relationships among individuals for each catchment (A) Wosera (B) Utu (C) Malala and (D) Mugil. Each coloured circle represents a haplotype (node), and black lines indicate shared alleles among individual haplotypes. The threshold for a connection was set at three matching loci between two haplotypes. [file 1475-2875-9-336-S7.PDF]
